# A Comparison of the Wellbeing of Orphans and Abandoned Children Ages 6–12 in Institutional and Community-Based Care Settings in 5 Less Wealthy Nations

**DOI:** 10.1371/journal.pone.0008169

**Published:** 2009-12-18

**Authors:** Kathryn Whetten, Jan Ostermann, Rachel A. Whetten, Brian W. Pence, Karen O'Donnell, Lynne C. Messer, Nathan M. Thielman, The Positive Outcomes for Orphans (POFO) Research Team

**Affiliations:** 1 Center for Health Policy, Duke Global Health Institute, Duke University, Durham, North Carolina, United States of America; 2 Terry Sanford Institute of Public Policy, Duke University, Durham, North Carolina, United States of America; 3 Departments of Psychiatry and Pediatrics, Duke University Medical Center, Durham, North Carolina, United States of America; 4 Center for Child and Family Health, Duke University, Durham, North Carolina, United States of America; 5 Department of Community and Family Medicine, Duke University, Durham, North Carolina, United States of America; 6 Department of Medicine, Division of Infectious Diseases and International Health, Duke University, Durham, North Carolina, United States of America; Penang Medical College, Malaysia

## Abstract

**Background:**

Leaders are struggling to care for the estimated 143,000,000 orphans and millions more abandoned children worldwide. Global policy makers are advocating that institution-living orphans and abandoned children (OAC) be moved as quickly as possible to a residential family setting and that institutional care be used as a last resort. This analysis tests the hypothesis that institutional care for OAC aged 6–12 is associated with worse health and wellbeing than community residential care using conservative two-tail tests.

**Methodology:**

The Positive Outcomes for Orphans (POFO) study employed two-stage random sampling survey methodology in 6 sites across 5 countries to identify 1,357 institution-living and 1,480 community-living OAC ages 6–12, 658 of whom were double-orphans or abandoned by both biological parents. Survey analytic techniques were used to compare cognitive functioning, emotion, behavior, physical health, and growth. Linear mixed-effects models were used to estimate the proportion of variability in child outcomes attributable to the study site, care setting, and child levels and institutional versus community care settings. Conservative analyses limited the community living children to double-orphans or abandoned children.

**Principal Findings:**

Health, emotional and cognitive functioning, and physical growth were no worse for institution-living than community-living OAC, and generally better than for community-living OAC cared for by persons other than a biological parent. Differences between study sites explained 2–23% of the total variability in child outcomes, while differences between care settings within sites explained 8–21%. Differences among children within care settings explained 64–87%. After adjusting for sites, age, and gender, institution vs. community-living explained only 0.3–7% of the variability in child outcomes.

**Conclusion:**

This study does not support the hypothesis that institutional care is systematically associated with poorer wellbeing than community care for OAC aged 6–12 in those countries facing the greatest OAC burden. Much greater variability among children within care settings was observed than among care settings type. Methodologically rigorous studies must be conducted in those countries facing the new OAC epidemic in order to understand which characteristics of care promote child wellbeing. Such characteristics may transcend the structural definitions of institutions or family homes.

## Introduction

Global, national and local leaders are struggling to find care solutions for the estimated 143,000,000 children worldwide who have had at least one parent die (hereafter defined as orphans) [1]. South and east Asia have the largest number of orphans (72,000,000) [2]; estimates for Africa indicate that 12% of all children on the continent will be orphaned by 2010. High mortality among young adults from conditions such as malaria, tuberculosis, pregnancy complications, HIV/AIDS and natural disasters are responsible for the large and increasing number of orphans [3]. A common demographic characteristic of orphans in the new epidemic across southern and eastern Africa is that rates of orphaning increase with age [4]. Millions more children are abandoned and in need of supportive living environments because their biological parents are not able to provide food, shelter and safety; are forced to leave their children to seek employment elsewhere; or are mentally or physically unable to care for children [2], [3]. The majority of OAC live in Sub-Saharan Africa and Southern and Southeastern Asia, in countries with rankings of medium and low on the 2009 Human Development Index (HDI).

Studies have demonstrated ill effects of being an orphaned or abandoned child (OAC) in resource poor countries, including traumatic grief, poverty, impaired cognitive and emotional development, less access to education and greater likelihood of being exploited as child labor [3], [5]–[11]. Other reports describe the challenges faced by families and communities in providing food, shelter, health care, and education for increasing numbers of OAC while the number of potential caregivers is diminishing due to increasing age-adjusted mortality [10], [12]–[15]. OAC are in need of living environments that promote their wellbeing.

Several influential studies have concluded that institutional care is damaging to the development of infants and small children relative to foster care [16]–[21]. One study of 65 children in the 1960s in London found that children placed in institutions who were then adopted or returned to their birth families (N = 39) did not suffer the negative emotional consequences that those left in institutions suffered [16], [17]. The Bucharest Early Intervention Project (BEIP) found that children 12 to 31 months of age in institutions in Romania, a high HDI country, had significantly higher rates of Reactive Attachment Disorder (RAD) and that RAD significantly decreased with increased quality of caregiving within the institutions [18]. Other studies in Romania found that young children in institutions were more likely to have RAD, cognitive delays, poorer physical growth and competence and negative behavior but that, within the same institution, when the ratio of children to caregivers was reduced over a 1 week period, the rates of RAD significantly decreased and that improving caregiving quality within an institution was associated with better outcomes [19], [20]. A meta-analysis of 42 studies conducted in 19 countries using IQ as an outcome found significant differences between the IQ of institutional children and those raised in family settings and that children younger at assessment and at age of being placed in the institution had worse outcomes than those who were either older or placed in the institution at an older age [21]. Significantly, in 3 of 4 medium or low HDI countries included no differences were found between the IQs of children in institutions and families [21]. These studies indicated that, at least in high and very high HDI countries, living in institutions is associated with poor outcomes, particularly for children aged 4 and younger; however, improving care in institutions improves outcomes. A limiting factor is the small number of institutions involved in the studies resulting in limited generalizability to institutions with different characteristics.

Other studies, primarily of children over age 4, show positive outcomes for institutionalized OAC under good caregiving and structural conditions [22]–[27]. For example, a study of orphanages in Eritrea found that children aged 9 to 14 in institutions with participatory decision making and where children were encouraged to become self-reliant had significantly fewer emotional and behavioral difficulties than children in institutions that did not have such characteristics [24], while another study found that changing the organizational structure of institutions so that they provided the children with greater decision making and encouragement resulted in improvements in child emotional wellbeing [25]. A study of orphanage alumni in the US found that the alumni fared well compared to their non-orphanage counterparts in terms of economic and emotional wellbeing and that alumni credited the structure of the orphanage, including the work ethic and religious teaching, with their long term wellbeing [27]. While provocative, study design flaws limit the generalizability of the later studies.

As the need for OAC care options increases particularly in medium and low HDI countries, global policies now recommend that one option, institutional care, be used as a last resort and that children in such care be moved to residential care as quickly as possible [28], [29]. These recommendations make explicit neither what constitutes an “institution” nor which characteristics of institutions are presumed to be responsible for poor OAC outcomes. They also do not recognize that in some cases, a family setting is either not an option or possibly a worse option than living in an institution that promotes child wellbeing. In the absence of such information, such policy movements limit care options without assurance that community environments will be more safe and supportive than the institutions from which children are moved.

This study uses cross-sectional data for children age 6 to 12 from the Positive Outcomes for Orphans (POFO) study to assess if the hypothesis that institutional care for children of this age group in countries facing the current OAC crisis is associated with poorer intellectual functioning, memory, emotion, behavior, and health than community care. The analyses describe the variation in child wellbeing of 1,357 children in 83 institutional care settings in 6 study sites across 5 medium HDI countries; these children are compared with 1,480 orphaned and abandoned community dwelling children from 311 community clusters (geographically bound sampling areas) in the same regions. All children included in the study had at least one parent who had died (83%) or had been left in the care of others (17%). Sensitivity analyses were conducted for subgroups of institution-based children and for 658 of the community dwelling children whose primary caregiver was not a biological parent. The variation in institutional care settings and child outcomes across and within community and institution-based care settings is examined.

This study adds to the body of evidence related to OAC caregiving in at least three ways. First, the study was conducted in six culturally, politically, religiously, historically and geographically distinct sites in 5 medium HDI nations facing rising OAC populations. Such a design reduces confounding between outcomes and culture. For example, in one culture extended families may traditionally care for the children of deceased siblings; in another culture such children may be shunned and treated harshly by extended families. Single country/culture studies could attribute differences related to cultural norms to the effects of the living structure. The structure of, and quality of caregiving in, the average institution in such places as Cambodia, Tanzania or Romania may be quite different from each other due to policy, religious, economic and cultural differences [30]–[35]. The same is true of family style care where, in addition, the quality of interaction is influenced by the cultural beliefs regarding acceptable treatment of OAC relative to biological children and the economic means of the family which may be less than those families caring for OAC in wealthier nations.

Second, this study attempted to draw a locally representative sample of institutions at each site resulting in one of the largest samples of institutions ever examined in any single study of OAC and perhaps the most representative of institutions at the sites. While studies comparing children living in one or two institutions to community-based children have explored a variety of community-based settings, they failed to consider the variability in institutional care.

Finally, this study focuses on children who are aged 6 to 12 and, while the results cannot be generalized to younger populations, this age group provides insight into the longer term effects of orphaning and the effects on children who were orphaned or abandoned at older ages; countries with emerging OAC epidemics have many children being orphaned at older ages. The magnitude of the OAC crisis demands that safe and sustainable care options be identified quickly and systematically.

## Materials and Methods

### Positive Outcomes for Orphans (POFO) Sampling

We employed two-stage random sampling survey methodology in 6 geographically defined regions of 5 less wealthy nations to identify a sample of 1,357 institution-living and 1,480 community-living OAC ages 6–12 who were statistically representative of the population of institution- and community-living OAC in those regions. The data collection was conducted between May 2006 and February 2008 among community-based and institution-based OAC and their caregivers. Four main instruments collected information from: 1) children reported to be aged 6 to 12 residing in communities who had a parent who had died or was missing; 2) children residing in institutions; 3) the children's primary caregivers; and 4) a person who could respond to administrative questions about the institution. Age inclusion criteria were based on survey instrument validity and pilot testing: The study sought to look at OAC aged 4 and older due to the findings of previous studies, but the pilot testing indicated that 4 and 5 year olds did not seem to understand many of the questions. Written informed consent was obtained from each participating caregiver and from the heads of participating institutions. Written assent was given by all participating children. Ethical approval was provided by the Duke University Institutional Review Board (IRB), the IRBs of Meahto Phum Ko'mah (Battambang, Cambodia), SaveLives Ethiopia (Addis Ababa, Ethiopia), Sharan (Delhi, India), ACE Africa (Bungoma, Kenya), and Kilimanjaro Christian Medical Centre (Moshi, Tanzania), and regulatory agencies in all participating countries: National Ethic Committee for Health Research (Cambodia), Ministry of Science and Technology (Ethiopia), Indian Council of Medical Research (India), Kenya Medical Research Institute (KEMRI), and the National Institute for Medical Research (Tanzania).

#### Country selection

From a group of 13 countries in which the research team had existing relationships with grassroots community organizations with an interest in the proposed research, five countries were selected that were culturally, historically, ethnically, religiously, politically, and geographically diverse from each other. Political boundaries were used to define six study areas *(See* *Table 1* *)*.

**Table 1:** Study enrollment and child characteristics.

	Inst. Sample		Comm. Sample	
*Site (N, %)*	Institutions	Children	Sampling Areas	Children
Cambodia	9 (11%)	157(12%)	47(15%)	250(17%)
Ethiopia	12(14%)	250(18%)	51(16%)	250(17%)
Hyderabad	14(17%)	250(18%)	51(16%)	250(17%)
Kenya	21(25%)	250(18%)	54(17%)	250(17%)
Nagaland	14(17%)	202(15%)	58(19%)	229(15%)
Tanzania	13(16%)	248(18%)	50(16%)	251(17%)
**Total**	83	1,357	311	1,480

*CHILD CHARACTERISTICS*		
**Age (Mean, SD)**	9.0 (1.8)	8.9 (1.8)
**Female (%)**	42.8	47.1

*♂ is father's status.*

♀ is mother's status.

*UK is Unknown.

#### Institution selection

For each of the six study areas, comprehensive lists of all institutions were created. To ensure broad representation, institutions were defined as structures with at least five orphaned children from at least two different families not biologically related to the caregiver(s). While this procedure could have resulted in the inclusion as “institutions” of family homes that are more like foster families, only 3 of the 83 institutions included were run out of caregivers' homes. Institutions specifically for street children, special needs children, and international adoption were excluded. The institutional sampling frame was generated through inquiries to local government officials, schools, and organizations working with orphans. Lists were randomized and institutions were approached sequentially until 250 children were enrolled into the study (see child selection below). If an institution refused participation, the next institution on the list was approached. To ensure that the sample was not dominated by large institutions, up to 20 children per institution were eligible to participate; at three sites this threshold was later eliminated to allow for the enrollment target of 250 children to be met at each site (see below). In total, 83 institutions participated in the study: 9 in Battambang (1 refusal), 12 in Addis Ababa (2 refusals), 13 in Kilimanjaro Region (1 refusal), 14 in Hyderabad (5 refusals), 14 in Dimapur and Kohima Districts of Nagaland (2 refusals), and 21 in Bungoma (no refusals). Reasons for refusals ranged from fear of psychological damage to the children to wanting monetary compensation for project participation (Appendix S4).

#### Selection of institution-based children

Each institution provided a list of all residential children under their care aged 6 to 12. Using a list of random numbers, up to 20 children per institution were randomly selected; the exception to this protocol was sites where the enrollment target of 250 children could not be met using this restriction: under this condition, all children in the age range became eligible to participate. Of the 5,243 children cared for by the institutions, 2,396 were reported to be age-eligible, and 1,357 were selected for enrollment. The number of participating children per institution ranged from 1 to 51. One quarter of children had been residing in the study institution for less than one year; 38% between one and three years; 21% between three and five years; and 10% more than five years. Information was missing for 6% of children. Five percent of children entered the institution before age 2; 15% at ages 2 to 4; 45% between ages 5 and 7; and 30% at ages 8 or above. These percentages only apply to study children. No information was collected on reasons for institutionalization or whether a child previously had spent time in other institutions.

#### Selection of community sampling areas

In each study area, the community sampling strategy involved the selection of 50 sampling areas (“clusters”) and 5 children per cluster. Geographic or administrative boundaries were used to define sampling areas: by necessity, the specific definition varied across sites. The primary community sampling aim was to select an unbiased sample of community-based care settings while adhering to the overarching methods.

#### Selection of community-based children

The definition of community-based children was an orphan, as defined above, not living in an institution; abandoned children living without either of their two parents were also eligible to participate. In each sampling area up to five eligible children were selected, either randomly from available lists, or through a house-to-house census conducted until 5 households with age-eligible children were identified. In 13 villages in Cambodia, 12 in Nagaland, and 1 in each of the remaining sites, substitutions for insufficient sampling areas or areas with fewer than five eligible children raised the number of children per sampling area to between 6 and 10. In households with multiple age-eligible children, one child was selected as the child whose first name started with the earliest letter in the alphabet. In total, 1,480 community-based children were enrolled in the study; 658 of these children were cared for by a primary caregiver other than the biological parent.

#### Caregiver selection

The children's (self-identified) primary caregivers were asked to respond to surveys about themselves and the children. In total, 193 institutional caregivers, ranging from 16 institutional caregivers in Nagaland to 52 in Cambodia, and 1,480 community-based caregivers participated in the assessments.

### Interviewer Training

One local male and female interviewer and a lead investigator from each site were trained on study protocol and procedures. A week-long training took place at a central location with all interviewers and primary investigators present. Following the training, the interviewers continued practicing and were certified only after repeated direct observation or video taping of interviews with local non-study children. The psychological testing was reviewed by the Duke child psychologist for fidelity to standard test procedures. Site visits, with interviewer observation, were conducted during the data collection to further ensure accuracy and consistency across interviewers and sites. Interviews were conducted in the child's residence and children were interviewed verbally in their native language.

### Measures

#### Subjective health

Caregiver-reported health measures included symptoms of fever, cough, and diarrhea in the last 2 weeks; general health of the child (single item from the Medical Outcomes Study Short Form 36 [36], with response options of “very good,” “good,” “fair,” “poor,” “very poor”); and physical wellbeing on the day of the interview.

#### Objective health growth

Growth measures included height and weight. Body Mass Index (BMI) and child height were age and gender standardized according to WHO growth charts [37].

#### Behavior and emotional health

The Strengths and Difficulties Questionnaire (SDQ) [38], [39], asked of children aged 11 and 12 and of the caregivers for all children, is a brief behavioral screening tool applicable for children 3–16 years old, used to assess behavioral and emotional difficulties and pro-social behavior. The SDQ has versions for parent, teacher, and self report. The five scales (emotional symptoms, conduct problems, hyperactivity/inattention; peer relationship, and pro-social behavior) have 5 items each; items are scored from 0–2. The first four scales result in the summary score of Total Difficulties, ranging from 0 to 40, with higher values signifying more difficulties. The raw Total Difficulties scores are used for group comparisons only.

The SDQ was selected because of the dimensions of behavior assessed, its brevity, the high correlations with well accepted but much longer child behavior measures [40], and its wide use in both resource rich and poor countries [41], [42]. One study reports SDQ differences between institutionalized and non-institutionalized children in the Netherlands, relating the findings to the low prevalence of secure attachment in the institutionalized group [43]. Although the SDQ has no published data regarding its psychometric properties or standardization in the five countries reported herein, its validity is supported by translation and use in 67 languages and the care with which translations and back translations are conducted in each of our study sites with native language speakers. In wealthy nations, mean scores range from 7.1 to 8.4 with scores indicating elevated (one standard deviation above the group mean) difficulties ranging from 12.8 to 14.3.

#### Cognitive development

Subtests from the Kaufman Assessment Battery for Children-II (KABC-II) [44] were used to evaluate the children's intellectual functioning. The KABC-II was chosen because it has been successfully utilized in low resource settings [45]; the visual attractiveness of the materials and tactile nature of the tests make them engaging for children around the world. Subtests appropriate for children ages 3 through 18 were used that can be administered with limited oral language, making them less dependent on language differences, and could be performed in less than 30 minutes. To assess sequential processing and short term memory through visual-motor abilities, spatial relations and visual motor integration, sustained attention, and visual problem solving abilities, 3 of the 5 subtests were chosen: Hand Movements, Triangles, and Pattern Reasoning. The scores reported here are the mean subtest scaled scores using the test's normative data for child age with a test result range from 0–19 with higher being better. The use of U.S. norms was justified because the scores were used to test group differences in an age-standardized way and not to assess individual child abilities.

The child's attention, motivation, and memory were assessed using a “Market List”, which is an adaptation of the California Verbal Learning Test (CVLT- Children's Version.) [46] The CVLT is used in a variety of settings to assess verbal learning and memory in children. The Market List was adapted to each site with the assistance of the local interviewers to reflect 15 items that would be seen in a local market, following the three semantic categories of the original CVLT. The child is read a list of items he/she might see in a market and asked to repeat the list. The items on the list were chosen to be common in everyday life in that area, even for a child who has not been to a local market. For this report, the score used for analysis was the mean of three administrations of the list.

### Analysis

Standard survey analytic techniques were used to estimate mean values of each outcome for institution-living OAC, community-living OAC, and community-living OAC not cared for by a biological parent, as well as 95% confidence intervals for the differences between means. Estimates accounted for unequal selection probabilities and the multilevel study design. Specifically, the survey estimation commands specified the stratified sampling by study site and the clustering of children within each institution or community cluster. For institution-living children, selection weights were defined as the inverse of the product of the sampling probabilities at the institution and child levels, and a finite population correction was applied in the calculation of the mean. For community-living children, sampling probabilities were not available since the sampling frame was not always known. In the calculation of means, the outcomes of institution-living OAC from each site were directly standardized to the age and gender distribution of that site's community-living OAC to reduce possible confounding by differences in the age or gender distributions between the community and institution-based samples.

To ensure robustness of the results, analyses were rerun on these subgroups: single orphans, double orphans, and single and double orphans only; ages 6–9 and 10–12; children in institutions with: <25 children, 50 or more children, and 100 or more children; children residing in their current living situation for: <1 year, 3 or more years and 5 or more years; and community children living with a biological parent.

In order to describe the proportion of total variation in outcomes that was attributable to each of the three levels of the survey design (study sites, care settings within sites, and individuals within care settings), we fit a linear mixed effects model (“model 1”) for each normally distributed outcome 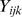 for child 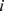 in care setting 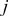 in study site 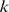, adjusting for age and gender and including random intercepts for sites 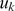 and care settings nested within sites 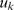; 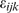 denotes child specific errors. The assumption of normally distributed residuals was checked with quantile (probit) plots [50].
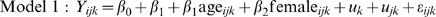



The variances of 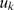, 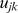 and 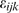, respectively, describe the variation in outcomes among study sites, variation among care settings within a site, and variation among individuals within a care setting.

To further describe the proportion of variability in outcomes, after adjustment for study site, age, and gender, that was attributable to overall differences between institutional and community-based care settings, we fit a second set of models that added fixed and random effects, 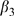 and 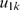, respectively, for a dichotomous variable indicating care setting type (“model 2”) [47].
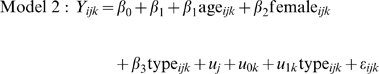



We estimated the proportion of variability attributable to care setting type 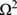 as
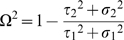
where *τ_i_* ^2^ and *σ_i_* ^2^ correspond to the care setting level variance and the individual level variance, respectively, estimated from models 1 and 2, respectively; Ω^2^ can be thought of as a partial R^2^ (conditional on age, gender, and site) within the context of a hierarchical model [48]–[49]. Analyses were conducted using Stata v.10.1 [51].

## Results

### Children

2,837 children participated in this study: 1,357 resided in institutional care settings and 1,480 in community-based care (Table 1). Females comprised 42.8% of institution-based children and 47.1% of community-based children; the average age was 9. The institutional sample is characterized by an age-related drop-off in the percentage of girls (p = 0.02; not shown): among 6-year olds, 47.4% of children were female, among children age 10 and older only 38.7% were female. This trend was the result of a site-specific drop in Hyderabad (p = 0.007) and was not observed in other sites or in community settings.

More than one-third of children in institutions (35.4%) and one in six children in the community (17.4%) were double orphans. Fifty-one percent of institution-based children and 76.8% of community-based children had one parent who was known to be alive. Fifty-five percent of community caregivers were biological parents; 22% were grandparents and 13% were aunts or uncles (not shown). Almost half of the children in institutions (47.6%) and one-third of children in the community (32.7%) had mothers who had died. Across settings, approximately 70% had fathers who had died.

### Institutions

**Figure 1:**
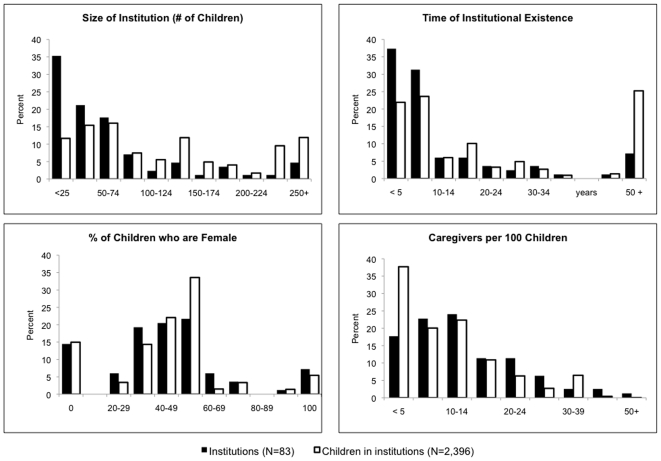
Characteristics of study institutions and distribution of children ages 6–12 residing in these institutions (N = 2,396). Legend: Dark bars describe the distribution of institutions. Light bars describe the distribution of institution-based children. Caregivers per 100 children calculated using the total number of children in the participating institutions.

Table 2 describes the variation in selected characteristics of participating institutions; Figure 1 illustrates this variation graphically, both across institutions and weighted by the number of children residing in these institutions. The mean (median) number of children in the institution was 63 (42); the mean (median) number of caregivers was 6.5 (4) and the mean (median) number of children per caregiver was 13.7 (9). The largest child-to-caregiver ratio for institutions with any children under age 2 was 16.9 (not shown). One quarter of the institutions (28.9%) had 20 or fewer children; the largest (17%) had 100 or more children (not shown). The largest institutions were located primarily in Addis Ababa and Hyderabad. One-third of the institutions had been in existence fewer than 5 years prior to the time of the interview; 31% were 5–9 years old, and 31% had been operating 10 years or more. Six institutions were all female and 11 all male.

**Table 2:** Characteristics of institutional care settings (N = 83) and caregivers in institutional and community Settings (N = 1,672).

*Institutional Characteristics (N = 83)*	Mean	SD	Median	Min	Max
**Number of children**	63.2	69.3	42	5	376
**Number of caregivers**	6.5	7.7	4	1	50
**Children per caregiver**	13.9	14.0	9.2	1	75.2
**Time of institutional existence**	**%**				
0–4 years	37.3				
5–9 years	31.3				
10+ years	31.3				

*Caregiver Characteristics (Institutions: N = 192; Community: N = 1,480)*		
	Institutions (N = 192)	Community (N = 1,480)
**Age (Mean, SD)**	35.5 (11.1)	41.6 (13.5)
**Female (%)**	77.3	83.9
**Education in years (Mean, SD)**	10.9 (4.2)	5.5 (4.3)
**Hours of work per week (Mean, SD)**	111.0 (55.4)	29.2 (23.9)
**Of those (%):**		
<20 hours	5.0	37.6
20–39 hours	8.3	26.3
40+ hours	50.0	36.2
residential (168 hours per week)	36.7	
**Earning an income (%):**		
in institution only	49.1	n/a
outside institution only	7.4	70.1
both inside and outside institution	18.3	n/a
none	25.1	29.9

### Caregivers

Three-quarters of institutional caregivers were female (77%), and the mean caregiver age was 35 (Table 2). On average, institutional caregivers had a 10^th^ grade education and worked more than 100 hours per week. Full-time residential work (168 hours per week) was reported by 37% of caregivers. One-third of the interviewed institutional caregivers reported working in the institutions without a salary (32.5%). Institutions reported providing room and board and a living stipend for many of the latter. Community caregivers, on average, were 42 years old, had a 5^th^ grade education, and worked less than full-time, on average, with 70% reporting earning an income.

### Child Characteristics

Caregivers subjectively rated the children's health on a five-point scale (higher = better); by these ratings, institutional-dwelling children had significantly better health scores than the community dwelling children (institution-living OAC: mean 4.00; community-living OAC: mean 3.72; weighted difference 0.34, 95% confidence interval [0.28, 0.41]) (Table 3). By caregiver report, institution-living children were also less likely to have had a cough, diarrhea, or fever in the two weeks before the interview (19.9 vs. 41.2%, weighted difference −20.6%, 95% CI [−24%,−18%]) or to be sick on the day of the interview (5.9% vs. 12.2%,), weighted difference −6.1%, 95% CI [−8%, −4%]). There were no differences between institution-living and community-living OAC in mean height for age or BMI for age. Total Difficulties scores on the Strengths and Difficulties questionnaire were lower (better) in institution-living than community-living OAC (weighted difference −0.78, 95% CI [−1.18, −0.38]). Institution-living OAC demonstrated greater intellectual functioning (weighted difference 0.38, 95% CI [0.25, 0.51]) and memory (weighted difference 0.59, 95% CI [0.40, 0.78]) than community-living OAC. In general, differences were more pronounced when comparing institution-based children with only community based children not cared for by their biological parents.

**Table 3:** Comparison of child outcomes between institutional and community-based care settings.

	Unweighted			Weighted^1^	
	Institutional children	All community children	Community children w/out bio. parents	Institution vs. community children	Institution vs. no biological parents
**Number of children**	1,357	1,480	658		
**Positive outcomes (higher is better)**	**Mean (SD)**	**Mean (SD)**	**Mean (SD)**	**Mean (CI)**	**Mean (CI)**
Caregiver-rated health	4.00 (0.76)	3.72 (0.83)	3.67 (0.83)	0.342 (0.28, 0.41)	0.367 (0.29, 0.44)
Height for age z score (WHO)	−0.96 (1.46)	−1.03 (1.29)	−1.10 (1.36)	0.011 (−0.08, 0.10)	0.074 (−0.04, 0.19)
BMI for age z score (WHO)	−0.68 (0.97)	−0.73 (1.39)	−0.84 (1.27)	0.072 (−0.01, 0.16)	0.113 (0.02, 0.21)
Cognition (K-ABC II)^2^	4.76 (1.89)	4.43 (1.71)	4.44 (1.83)	0.379 (0.25, 0.51)	0.429 (0.28, 0.58)
California Verbal Learning Test^3^	7.77 (2.35)	7.22 (2.24)	7.29 (2.24)	0.590 (0.40, 0.78)	0.599 (0.38, 0.82)
S&D Total Difficulties score (0 = worst, 40 = best)	10.13 (6.07)	10.93 (5.66)	11.05 (5.84)	−0.778 (−1.18, −0.38)	−0.968 (−1.48, −0.46)
**Negative outcomes (higher is worse)**	**N (%)**	**N (%)**	**N (%)**	**% (CI)**	**% (CI)**
Diarrhea/fever/cough in last 2 weeks	269 (19.9)	603 (41.2)	273 (41.5)	−20.6 (−0.24, −0.18)	−20.4 (−0.24, −0.16)
Child sick on day of caregiver interview	79 (5.9)	179 (12.2)	69 (10.4)	−6.1 (−0.08, −0.04)	−4.5 (−0.07, −0.02)

1 Weighted means and standard errors account for sampling weights and the complex survey design and are further adjusted for age and gender (standardized to the site-specific distribution of age and gender among community children).

2 Mean of three K-ABC-II subtests with responses converted to scaled scores using age-specific norms (range 0–19 with higher being better) distribution of age and gender among community children).

3 CVLT score defined as the mean number of items recalled in three administrations (range 0–15).

**Figure 2:**
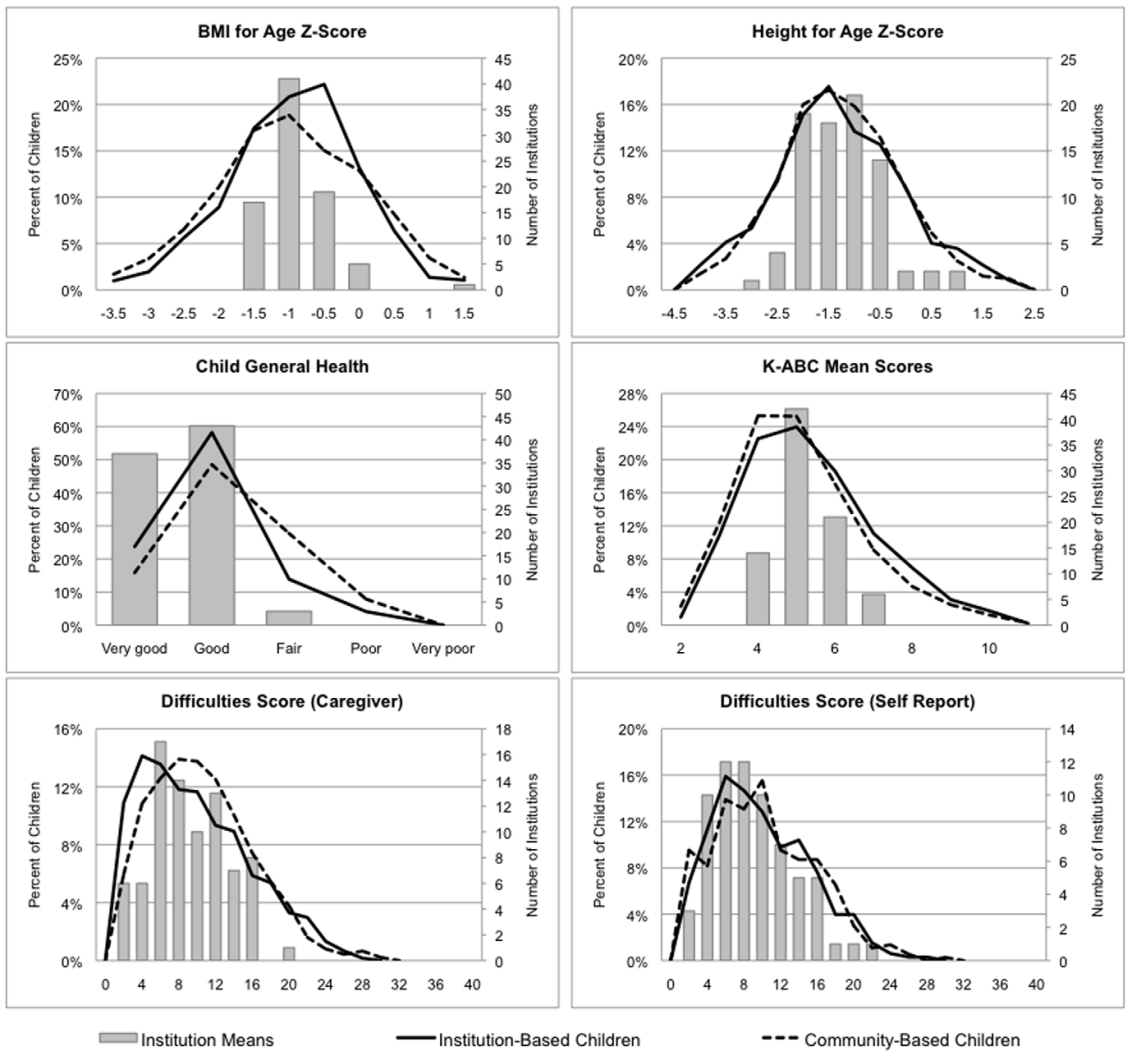
Distribution of child outcomes for community-based (N = 1,480) and institution-based (N = 1,357) children residing in 83 institutions. Legend: Grey bars describe the distribution of institution means. Solid line describes the distribution of child outcomes among institution-based children. Dotted line describes the distribution of child outcomes among community-based children.

There was substantial variation in mean child outcomes among participating institutions, and even greater variation in outcomes across institution-based children (Figure 2). The distribution of child outcomes among institution-based children was similar to that of study children in residing in communities.

After adjustment for age and gender, differences between study sites accounted for 2.2% to 22.5% of the variation in child outcome measures, while differences between care settings within sites accounted for 7.9–13.9% of the total variation and differences between individuals within care settings accounted for 63.6%–86.8% (Table 4). Differences between care settings within sites accounted for similar proportions of total variation whether considering only institution-living OAC (5.9–21.2%) or community-living OAC (1.8–17.1%). In the models that conditioned on age, gender, and site, the dichotomous variable for care setting type (institution vs. community-based) explained 0.3–6.9% of the total variation in child outcomes.

**Table 4:** Percent of total variation in outcomes attributable to differences among sites, care settings and individuals, and explained by care setting type.

	Variation attributable to differences among^1^			
	Sites	Care settings within sites	Individuals within care settings	Variation explained by care setting type^3^
**Health**	7.0	21.3	71.7	3.8
**Height for age z score (WHO)**	5.4	7.9	86.8	0.9
**BMI for age z score (WHO)**	14.3	13.4	72.3	6.9
**SDQ Total Difficulties Score**	22.5	13.9	63.6	0.3
**Cognition (K-ABC-II scores)** 4	4.0	10.1	85.9	1.8
**California Verbal Learning Test** 5	2.2	12.1	85.7	2.8

1 From a linear mixed model adjusted for age and gender and including random effects for sites and care settings.

2 Institutions or community clusters sampled within sites.

3 Percent reduction in overall variance upon introduction of dichotomous variable and random site-level slopes for setting type, conditional on site, age, and gender.

4 Mean of three K-ABC-II subtests with responses converted to scaled scores using age-specific norms (range 0–19 with higher being better).

5 CVLT score defined as the mean number of items recalled in three administrations (range 0–15).

Our sensitivity analyses of subgroups (e.g., excluding non-orphaned children, including only single orphans, only double orphans, only children in their current setting less than 1 year and alternatively only 5 years and longer, and only children in small (25 or less) or large (100 or more) institutions) did not change the overall results of the analyses (Appendixes S1, S2, S3). The differences in cognition and memory remained significant in all analyses, the biometric health measures became significant in the direction of better health for children in institutions and behavior became insignificant while still trending toward better behavior for children in institutions. In general, the results were consistent in direction and magnitude.

## Discussion

These analyses were designed to test the hypothesis that institutional care for OAC aged 6–12 is associated with worse child health and wellbeing than community care, specifically in areas of the world most affected by the current orphan crisis and where many children are orphaned at a later age. The results do not support this hypothesis. While it is possible that respondent bias accounts for better subjective health scores for children in institutions, the lack of significant differences on the biometric scores and the lower prevalence of recent illness suggest that the growth and overall health of children in the institutions is no worse than that of children in communities. The institution-based children scored higher on intellectual functioning and memory and had fewer social and emotional difficulties. The differences were more pronounced when comparing these children only to community-based children not cared for by a biological parent. Results were robust in the sensitivity analyses. There were children in the study who scored poorly across all dimensions while others scored highly; this variation was equally true for children in institutions and communities. These findings challenge the policy recommendations to use institutions, for all children, only as a last resort and to get children who have to be placed in institutions back out to family-style homes as quickly as possible [52]. There is even a movement to evaluate the success of institutions by how quickly they get the children back out to family-style homes [53]. The evaluation measures would likely affect future funding of the institution and therefore provide an adverse incentive to send children out to family-style homes that may not be able to provide adequate care to promote the child's wellbeing.

The similarity of distributions in child wellbeing in community and institution-based children suggests that ‘institutional care,’ per se, should not be categorically described as damaging or inappropriate for all children. Relative to variations in child outcomes within communities and within institutions, and between care settings of each type, the overall differences between communities and institutions were small. There was significant variation in average child wellbeing across institutions and across community settings, explaining more of the variation in child outcomes than differences between institution- and community-based care settings.

Institutions varied across many dimensions, including the number of children and the gender distribution of the children they housed, including all female, all male and mixed institutions. They varied by the length of time that they had been in operation, and by the characteristics of the caregivers. Such differences may be important determinants of child outcomes and should be further explored. There was also significant variation in child wellbeing in community settings. Advocating the moving of children from one care structure to another, such as from institutions to community settings, without understanding the causes of the differences in child outcomes may place children at risk of worse outcomes.

A potentially important finding of this study is that is that, on average, the institutions look quite different from institutions included in most of the previous studies that compared the outcomes of children in institutions and those in community settings. For example, simply the finding that many of the caregivers live at the institutions, work long hours and may be paid only in room and board is important. This supports a statement made by a medical student from Uganda who was orphanded, that “what people do not realize is that this [the institution] is our community response [54].” Many institutions grew out of the community to meet the need of caring for the new wave of orphans and are a part of the community in a way that institutions in other regions and perhaps of the past were not. These institutions are not family-style/community care and they are not foster care, but they also do not look like institutions as we have come to think of them. If this represents a new kind of care structure that minimizes some of the damage to children demonstrated in past studies and in different contexts, then researchers and policy makers need to: 1) gain a better understanding of these organic care structures and 2) ensure that they are not hindered by blanket policies about institutions.

Children entering institutions are likely to differ systematically from orphans cared for in their communities. Indicators of such bias in this study are the greater proportion of institution-based children that were double-orphans, and maternal death being a greater risk factor for being in an institution than paternal death. Systematic biases resulting from past life events will influence children's longer term outcomes and may be reflected in cross-sectional differences between institution-based and community-based children. For example, children in institutions may have experienced the orphaning or abandonment at a later age, when they are less vulnerable, relative to the children in the community. Many environmental influences on health and wellbeing are cumulative, the subject of substantial lag times, and will differ by the dimensions of wellbeing (e.g., growth, emotion, behavior and cognition). Cross-sectional analyses, such as the one presented here, cannot account for these effects. Similarly, the study does not inform us as to why there are fewer older female children at one site; one might speculate that they were hired or forced into domestic work or prostitution, but only longitudinal studies will allow researchers to consider such speculations. Longitudinal studies will further advance our knowledge as to the particular care characteristics that best support children in their emotional, intellectual and physical development.

The results of this analysis cast doubt on the generalizability of past studies indicating that institutions are systematically associated with poor child outcomes to children of this age group, 6 to 12 years of age, in less wealthy nations. The differences in the study findings may be due to several causes. For example: This study is of older children and cannot be generalized to other age groups, particularly the very young where much of the strong evidence demonstrating the detrimental effects of institutions on child brain development has been found. It is possible that the negative effects of institutions that have been found in past studies either do not hold for older children, or that measurements need to be more precise to find differences.

Secondly, the countries included may have poorer community settings where caregivers are not able to provide as adequate care. It is possible that when communities are very poor, as indicated by the HDI scores for the sites included in this study [55], that differences between institutional care and family-style care are minimized. In such places, positive institutions may provide a place where children can focus on education and their own needs rather than supporting their families. If the latter is true, then it may not be that institutional care is “good,” but that it is better than the community alternative. Further, the study results cannot be generalized to wealthier areas where orphaning and institutions are more rare.

Finally, cultures may differ so that institutional caregivers provide more parent-like support; and children living in the institutions may be more incorporated into the surrounding community. Because of their lack of visibility, intensive effort was required to create the sampling frames from which institutions were sampled at each site. Small locally run institutions were hardest to locate. The virtual invisibility of a majority of institutions in less wealthy nations may be one reason why the results of this study contradict those reported in previous studies. It may be that locally run institutions have characteristics that are more conducive to positive child outcomes than the more formal and visible institutions that have typically been assessed in OAC-related research.

As the number of OAC increases in medium and low HDI countries, it is vital not to discount an important care structure before conclusively assessing whether these structures have systematic negative impacts on the millions of children for which they care. This study indicates that in these culturally diverse medium HDI nations, OAC aged 6–12 cared for in institutionalized settings had outcomes that are as good and as poor as their community-based counterparts. While there was great variation in child wellbeing across outcome measures, this variation was not determined by residence in one physical structure over another. This study argues for a move beyond the dichotomized choice set of community vs. institution-based care towards an analysis of the specific characteristics of these care settings which are associated with improved child outcomes. Future studies that seek to assist medium and low HDI countries in finding feasible solutions for their OAC need to be conducted with rigorous methods in these countries.

## Supporting Information

Appendix S1 Differences in child outcomes between institutional and community-based care settings. Institutional sample stratified by children's time spent in the current institutional care setting
(0.12 MB DOC)

Appendix S2 Comparison of child outcomes between institutional and community-based care settings. Institutional sample stratified by children's age at entry into the current institutional care setting
(0.04 MB DOC)

Appendix S3 Comparison of child outcomes between institutional and community-based care settings. Institutional sample stratified by size of institutional care setting.
(0.04 MB DOC)

Appendix S4 Reasons for Institutional Study Refusals
(0.03 MB DOC)
