# Supplementary material for: A Comparison of the Wellbeing of Orphans and Abandoned Children Ages 6–12 in Institutional and Community-Based Care Settings in 5 Less Wealthy Nations
Source: PLoS One. 2009 Dec 18;4(12):e8169. doi: 10.1371/journal.pone.0008169 (PMC2790618; doi:10.1371/journal.pone.0008169)
Supplement: Appendix S1 — Differences in child outcomes between institutional and community-based care settings. Institutional sample stratified by children's time spent in the current institutional care setting (0.12 MB DOC) [file pone.0008169.s001.doc]

| **Appendix S1. Differences in child outcomes between institutional and community-based care settings.**  Institutional sample stratified by children’s time spent in the current institutional care setting | | | | |  |
| --- | --- | --- | --- | --- | --- |
|  |  |  |  |  |  |
|  |  |  | Weighted differences in means or proportions^1,2^  (confidence intervals of differences in parentheses) | | |
|  |  |  |  |  |  |
| Time spent in the current institutional care setting |  | < 1 year | 1 to <3 years | 3 to 5 years | >5 years |
|  |  |  |  |  |  |
| Number of institution-based children^3^ |  | 330 | 515 | 276 | 116 |
|  |  |  |  |  |  |
| *Positive outcomes (higher score is better)* |  |  |  |  |  |
| Caregiver-rated health |  | 0.385 (0.32, 0.45) | 0.331 (0.27, 0.39) | 0.335 (0.26, 0.41) | 0.464 (0.37, 0.56) |
| Height for age z score (WHO) |  | 0.026 (-0.05, 0.10) | -0.086 (-0.18, 0.01) | 0.121 (0.01, 0.23) | 0.236 (0.11, 0.36) |
| BMI for age z score (WHO) |  | -0.007 (-0.09, 0.08) | 0.054 (-0.04, 0.15) | 0.079 (-0.01, 0.16) | 0.284 (0.20, 0.37) |
| Cognition (K-ABC II) 3 |  | 0.166 (0.03, 0.30) | 0.19 (0.06, 0.32) | 0.277 (0.14, 0.42) | 0.449 (0.32, 0.58) |
| California Verbal Learning Test |  | 0.572 (0.42, 0.72) | 0.354 (0.16, 0.54) | 0.972 (0.79, 1.15) | 0.667 (0.50, 0.83) |
|  |  |  |  |  |  |
| *Negative outcomes (higher score or percentage is worse)* | | |  |  |  |
| Diarrhea/Fever/Cough in last 2 weeks |  | -21.6% (-0.25, -0.19) | -23.0% (-0.26, -0.20) | -16.8% (-0.20, -0.14) | -29.8% (-0.33, -0.27) |
| Child sick on day of caregiver interview |  | -4.3% (-0.07, -0.02) | -7.5% (-0.10, -0.05) | -3.0% (-0.05, -0.01) | -8.2% (-0.10, -0.07) |
| S&D Total Difficulties Score (0=worst, 40=best) |  | -1.607 (-2.03, -1.19) | -0.256 (-0.69, 0.18) | -0.81 (-1.23, -0.39) | -0.666 (-1.10, -0.23) |
|  |  |  |  |  |  |
|  |  |  |  |  |  |
| ^1^ Differences relative to 1,480 orphaned and abandoned children residing in community settings | | | |  |  |
| ^2^ Differences in means and confidence intervals (in parentheses) account for sampling weights and the complex survey design | | | | | |
| and are adjusted for age and gender (standardized to the site-specific distribution of age and gender among community children) | | | | | |
| ^3^ 120 children excluded due to missing information on time in institutional care (79), or single observations per sub-stratum in variance estimation (41) | | | | | |
