# Supplementary material for: A Comparison of the Wellbeing of Orphans and Abandoned Children Ages 6–12 in Institutional and Community-Based Care Settings in 5 Less Wealthy Nations
Source: PLoS One. 2009 Dec 18;4(12):e8169. doi: 10.1371/journal.pone.0008169 (PMC2790618; doi:10.1371/journal.pone.0008169)
Supplement: Appendix S2 — Comparison of child outcomes between institutional and community-based care settings. Institutional sample stratified by children's age at entry into the current institutional care setting (0.04 MB DOC) [file pone.0008169.s002.doc]

| **Appendix S2. Comparison of child outcomes between institutional and community-based care settings.**  Institutional sample stratified by children’s age at entry into the current institutional care setting | | | | | | | | | |  | |  |
| --- | --- | --- | --- | --- | --- | --- | --- | --- | --- | --- | --- | --- |
|  |  | |  | |  | | | | |  | |  |
|  |  | | Weighted differences in means or proportions^1,2^  (confidence intervals of differences in parentheses) | | | | | | | | |  |
|  |  | |  | |  | | | | |  | |  |
| Age at entry into institution |  | | Before age 5 | | Age 5-7 | | | | | Age 8 or older | |  |
|  |  | |  | |  | | | | |  | |  |
| Number of institution-based children^3^ |  | | 249 | | 597 | | | | | 401 | |  |
|  |  | |  | |  | | | | |  | |  |
| *Positive outcomes (higher score is better)* |  | |  | | |  | |  | | | |  |
| Caregiver-rated health |  | | 0.37 (0.30, 0.44) | | | 0.347 (0.28, 0.42) | | 0.281 (0.22, 0.35) | | | |  |
| Height for age z score (WHO) |  | | 0.216 (0.11, 0.32) | | | 0.041 (-0.06, 0.14) | | -0.262 (-0.38, -0.14) | | | |  |
| BMI for age z score (WHO) |  | | -0.031 (-0.12, 0.05) | | | 0.067 (-0.03, 0.16) | | 0.069 (-0.02, 0.16) | | | |  |
| Cognition (K-ABC II) 3 |  | | 0.536 (0.42, 0.66) | | | 0.355 (0.22, 0.49) | | -0.138 (-0.31, 0.03) | | | |  |
| California Verbal Learning Test |  | | 0.466 (0.32, 0.61) | | | 0.730 (0.55, 0.91) | | 0.473 (0.27, 0.68) | | | |  |
|  |  | |  | | |  | |  | | | |  |
| *Negative outcomes (higher score or percentage is worse)* | | | | | |  | |  | | | |  |
| Diarrhea/Fever/Cough in last 2 weeks | |  | | -23.3% (-0.26, -0.20) | | | -21.4% (-0.25, -0.18) | | -21.9% (-0.26, -0.18) | | |  |
| Child sick on day of caregiver interview | |  | | -6.9% (-0.09, -0.05) | | | -3.7% (-0.06, -0.02) | | -5.1% (-0.07, -0.03) | | |  |
| S&D Total Difficulties Score (0=worst, 40=best) | |  | | -0.229 (-0.62, 0.16) | | | -0.883 (-1.32, -0.45) | | -0.531 (-1.00, -0.07) | | |  |
|  |  | |  | | |  | | | | |  | |
|  |  | |  | |  | | | | |  | |  |
| ^1^ Relative to 1,480 orphaned and abandoned children residing in community settings | | | | | | | | | |  | |  |
| ^2^ Differences in means and confidence intervals (in parentheses) account for sampling weights and the complex survey design | | | | | | | | | | | |  |
| and are adjusted for age and gender (standardized to the site-specific distribution of age and gender among community children) | | | | | | | | | | | |  |
| ^3^ 110 children excluded due to missing information on time in institutional care (79), or single observations per sub-stratum (31) | | | | | | | | | | | |  |
