# Supplementary material for: A Comparison of the Wellbeing of Orphans and Abandoned Children Ages 6–12 in Institutional and Community-Based Care Settings in 5 Less Wealthy Nations
Source: PLoS One. 2009 Dec 18;4(12):e8169. doi: 10.1371/journal.pone.0008169 (PMC2790618; doi:10.1371/journal.pone.0008169)
Supplement: Appendix S3 — Comparison of child outcomes between institutional and community-based care settings. Institutional sample stratified by size of institutional care setting. (0.04 MB DOC) [file pone.0008169.s003.doc]

| **Appendix S3. Comparison of child outcomes between institutional and community-based care settings.**  Institutional sample stratified by size of institutional care setting | | | | |  |
| --- | --- | --- | --- | --- | --- |
|  |  |  |  |  |  |
|  |  |  | Weighted differences in means or proportions^1,2^  (confidence intervals of differences in parentheses) | | |
|  |  |  |  |  |  |
| Number of children in institutional care setting |  | <25 | 25-49 | 50-99 | 100+ |
|  |  |  |  |  |  |
| Number of institution-based study children^3^ |  | 285 | 244 | 464 | 245 |
|  |  |  |  |  |  |
| *Positive outcomes (higher score is better)* |  |  |  |  |  |
| Caregiver-rated health |  | 0.196 (0.08, 0.31) | 0.111 (0.04, 0.18) | 0.385 (0.32, 0.45) | 0.42 (0.31, 0.53) |
| Height for age z score (WHO) |  | 0.156 (0.05, 0.27) | 0.052 (-0.06, 0.16) | 0.021 (-0.08, 0.12) | 0.164 (-0.02, 0.35) |
| BMI for age z score (WHO) |  | 0.088 (0.01, 0.17) | 0.013 (-0.09, 0.11) | 0.057 (-0.03, 0.14) | -0.075 (-0.21, 0.06) |
| Cognition (K-ABC II) 3 |  | 0.515 (0.40, 0.63) | 0.494 (0.34, 0.64) | 0.096 (-0.02, 0.21) | 0.84 (0.61, 1.07) |
| California Verbal Learning Test |  | 0.617 (0.45, 0.79) | 0.903 (0.70, 1.11) | 0.526 (0.36, 0.69) | 0.702 (0.21, 1.20) |
|  |  |  |  |  |  |
| *Negative outcomes (higher score or percentage is worse)* | | |  |  |  |
| Diarrhea/Fever/Cough in last 2 weeks |  | -21.1% (-0.24, -0.18) | -19.0% (-0.24, -0.14) | -25.1% (-0.28, -0.22) | -29.7% (-0.35, -0.25) |
| Child sick on day of caregiver interview |  | -9.3% (-0.11, -0.08) | -4.2% (-0.08, -0.01) | -8.3% (-0.10, -0.06) | -8.0% (-0.11, -0.05) |
| S&D Total Difficulties Score (0=worst, 40=best) |  | -1.776 (-3.06, -0.49) | -1.23 (-1.76, -0.70) | -0.474 (-0.94, -0.01) | 0.201 (-0.47, 0.87) |
|  |  |  |  |  |  |
|  |  |  |  |  |  |
| ^1^ Differences relative to 1,480 orphaned and abandoned children residing in community settings | | | |  |  |
| ^2^ Differences in means and confidence intervals (in parentheses) account for sampling weights and the complex survey design | | | | | |
| and are adjusted for age and gender (standardized to the site-specific distribution of age and gender among community children) | | | | | |
| ^3^ 119 children excluded due to single observations per sub-stratum in variance estimation | | | | | |
