# Supplementary material for: A Comparison of the Wellbeing of Orphans and Abandoned Children Ages 6–12 in Institutional and Community-Based Care Settings in 5 Less Wealthy Nations
Source: PLoS One. 2009 Dec 18;4(12):e8169. doi: 10.1371/journal.pone.0008169 (PMC2790618; doi:10.1371/journal.pone.0008169)
Supplement: Appendix S4 — Reasons for Institutional Study Refusals (0.03 MB DOC) [file pone.0008169.s004.doc]

**Appendix S4: Reasons for Institutional Study Refusals**

| The following were reasons for refusals from the 11 institutions: |
| --- |
| Fear that the study would psychologically damage the children (Tanzania) |
| The institution wanted financial compensation for study participation (Cambodia, Hyderabad, Nagaland) |
| The institution reported that many interviewers (university students, etc.) had already asked the children  about their lives and the director felt that the children are psychologically damaged by these studies (Ethiopia) |
| The manager was abroad at that time and no one was there to decide (Ethiopia) |
| Fear that the study was designed to prove that institutions are bad (Hyderabad) |
| Wanted to protect the confidentiality of the children in an institution for rescued sex workers and sexual abuse victims (Hyderabad) |
| Other researchers were doing studies with their children so they did not have time for another study (Hyderabad) |
| Stated that they could not participate without ministry approval (Hyderabad) |
| Institution was run by the an insurgent group and did not allow visitors due to security issues (Nagaland) |
